# The Role of Promoter-Associated Histone Acetylation of *Haem Oxygenase-1* (*HO-1*) and *Giberellic Acid-Stimulated Like-1* (*GSL-1*) Genes in Heat-Induced Lateral Root Primordium Inhibition in Maize

**DOI:** 10.3389/fpls.2018.01520

**Published:** 2018-10-16

**Authors:** Hao Zhang, Mengxia Yue, Xueke Zheng, Mayank Gautam, Shibin He, Lijia Li

**Affiliations:** ^1^State Key Laboratory of Hybrid Rice, College of Life Sciences, Wuhan University, Wuhan, China; ^2^State Key Laboratory of Cotton Biology, College of Life Sciences, Henan University, Kaifeng, China

**Keywords:** *Zea mays*, heat stress, epigenetics, histone acetylation, HDACs, H3K9, H4K5, lateral root primordium inhibition

## Abstract

In plants, lateral roots play a crucial role in the uptake of water and nutrients. Several genes such as *Zea mays Haem Oxygenase-1* (*ZmHO-1*) and *Giberellic Acid-Stimulated Like-1* (*ZmGSL-1*) have been found to be involved in lateral root development. In the present investigation, we observed that heat treatment might be involved in the inhibition of lateral root primordium (LRP) formation in maize, accompanied by an increase in global acetylation levels of histone 3 lysine residue 9 (H3K9) and histone 4 lysine residue 5 (H4K5), suggesting that histone modification was related to LRP inhibition. However, Trichostatin A (TSA), an inhibitor of histone deacetylases (HDACs), apparently did not inhibit the LRP formation, revealing that global hyperacetylation might not be the determining factor in the LRP inhibition induced by heat stress. Furthermore, expression of genes related to lateral root development in maize, *ZmHO-1* and *ZmGSL-1*, was down-regulated and the acetylation levels in the promoter region of these two genes were decreased under heat stress, suggesting that promoter-associated histone acetylation might be associated with the expression of *ZmHO-1* and *ZmGSL-1* genes which were found to be involved in the heat-induced LRP inhibition in maize.

## Introduction

Plant roots play a crucial role in communication at the root-soil interface (i.e., water and nutrition uptake), and anchorage (Villordon et al., 2014). Root system architecture (RSA) is regulated by an endogenous genetic program, including cultivars or inbred lines (Song et al., 2016) and external factors, including biotic and abiotic environment (Gifford et al., 2013; Dondoni and Marra, 2014; Zhang et al., 2014; Yu et al., 2015). A typical plant root is divided into several functional zones along the root’s longitudinal axis, including the root cap, meristem zone, elongation zone, mature zone, and lateral root zone (Wilson et al., 2015).

Maize (*Zea mays*), an agronomically important cereal crop, plays an important role in food, animal feed, and biofuel production worldwide and its roots indicate spatio-temporal complexity and have distinct genetic control (Smith and De Smet, 2012; Rogers and Benfey, 2015; Tai et al., 2016; Song et al., 2016). Morphology of maize roots consists of embryonic root system: an embryonic primary root forms 2 or 3 days after germination and a variable number of seminal roots arise a week after germination and extensive post-embryonic shoot-borne root system: crown roots arise from consecutive underground nodes of the stem which initiates ∼10 days after germination, and brace roots arise from consecutive aboveground nodes of the stem which initiates ∼6 weeks after germination (Hochholdinger et al., 2004; Zhang et al., 2014; Tai et al., 2016). Lateral root formation can be characterized into two major phases: pericycle activation (stimulation and dedifferentiation of pericycle cells) which proliferates to form a LRP and meristem establishment which occurs via cell expansion and activation of the lateral root meristem (Malamy and Benfey, 1997; De Smet, 2012; Zhang et al., 2014). Lateral roots arise from embryonic primary and seminal roots and post-embryonic shoot-borne roots, constituting an extensive underground branching network which increases the interacting surface (Malamy, 2005; Hochholdinger and Zimmermann, 2008; Rogers and Benfey, 2015).

It has been reported that maize lateral roots arise via the division of pericycle and endodermis cell (Fahn, 1990; Yu et al., 2016), whereas *Arabidopsis* lateral roots arise completely from pericycle cells (Dubrovsky et al., 2000; Beeckman et al., 2001). Lateral root formation is affected by various intrinsic and extrinsic factors, such as phytohormones and environmental stimuli (Marhavý et al., 2016; Yu et al., 2016). Substantial evidence has revealed that lateral root initiation is regulated by alteration of cyclins and cyclin-dependent kinases (CDKs) induced by auxin in pericycle cells (Malamy, 2005; Yu et al., 2015). Several related genes have been found to be associated with the lateral root initiation and formation in plants. Members of the plant-specific *Gibberellic Acid-stimulated Arabidopsis* (*GASA*) gene family play a significant roles in diverse biological processes, such as seed germination (Rubinovich and Weiss, 2010; Zhong et al., 2015) and flower induction (Roxrud et al., 2007). *GAST1* (*Gibberellic Acid Stimulated Transcript 1*) is the first member of the *GASA* gene family and has been identified in tomato which has a similar function in lateral root formation to *Root System Induced1* (*RSI1*) (Shi et al., 1992). The *GASA* gene family consists of 14 genes; evidence on *GASA4* has demonstrated that it expressed significantly in meristematic tissue namely, primary and lateral roots (Aubert et al., 1998). Several studies have suggested that *GASA*-like genes mainly regulated cell division and elongation (Ben-Nissan et al., 2004). *Gibberellic Acid Induced Petunia 1* (*GIP1*) identified from petunia (*Petunia hybrida*) has been suggested to be closely related to stem elongation. While *GIP4* and *GIP5* have shown similar expression changes during cell division regulation as *GASA4* in *Arabidopsis* (Aubert et al., 1998; Ben-Nissan et al., 2004). Ten members of *GASA*-like family have been identified in maize (Zimmermann et al., 2010). The *Z*. *mays Gibberellic Acid- Stimulated Like* (*ZmGSL*) family encodes small proteins of 75 to 128 amino acids which contain 12 perfectly conserved cysteines (Zimmermann et al., 2010). The result from lateral root mutants *lrt1* and *rum1* has indicated that *ZmGSL2*, *ZmGSL4*, *ZmGSL6*, and *ZmGSL9* contributed to lateral root formation and development (Zimmermann et al., 2010). Specifically, *ZmGSL2* represented a maize-specific checkpoint of lateral root formation in primary roots, while *ZmGSL4*, *ZmGSL6*, and *ZmGSL9* is likely to contribute to the initial events of lateral root formation. Recently, it was found that *Haem oxygenase* (*HO*) gene also acts as an important regulator in the lateral root formation (Han et al., 2012). HO catabolizes haem into three products: carbon monoxide (CO), biliverdin (BV) and free iron (Hsu et al., 2013). Three HO proteins, HO-1, HO-2, and HO-3, have been identified in mammals (Maines, 2004). However, several forms of *HO*-*1*-like genes have been isolated and identified from *Arabidopsis* (*AtHO-1*), Tomato (*LeHO-1*) and rice (*OsHO-1*) (Han et al., 2012; Meng and Liao, 2016; Mahawar and Shekhawat, 2018). The expression of *HO-1* can be induced by multiple stimuli, such as, reactive oxygen species (ROS) (Chen et al., 2009), salinity or heavy metal stress (Han et al., 2008; Xie et al., 2008), and ultraviolet radiation (Yannarelli et al., 2006). Hence, it is well established that the expression of *HO-1* is an antioxidant mechanism against oxidative damage subject to diverse stress (Shekhawat and Verma, 2010). In maize, ZmHO-1 has a conserved HO signature sequence and shares highest homology with rice OsHO-1 protein. It is established that up-regulation of *ZmHO-1* is closely associated with maize lateral root formation by modulating cell cycle regulatory genes and enhancing CO production (Han et al., 2012).

Histone post-translational modification can occur at the N-terminal tail of core histones (H2A, H2B, H3, H4) through acetylation, methylation, phosphorylation, and ubiquitination (Jenuwein and Allis, 2001). Previous studies have shown that histone acetylation are involved in plant resistance response to abiotic stimuli (Hu et al., 2011; Zhao et al., 2014). Here, we illustrated that heat stress might led to the inhibition of LRP formation in maize. To establish the role of epigenetic mechanisms in regulating LRP formation in maize, we have studied the role of TSA, an inhibitor of HDACs. Our findings revealed that TSA did not inhibit lateral root formation, suggesting that global hyperacetylation might not be the determining factor regulating inhibition of lateral root formation in maize. Furthermore, we have analyzed the role of lateral root initiation/development genes, such as *ZmHO-1* and *ZmGSL-1* genes under heat stress. Our research corroborates that *ZmHO-1* and *ZmGSL-1* genes showed down-regulation and acetylation of the promoter regions of these two genes was decreased under heat stress, indicating that promoter-associated histone acetylation of *HO*-*1* and *GSL*-*1* genes might be involved in the heat-induced LRP inhibition in maize.

## Results

### Heat Stress Inhibits Lateral Root Formation

**FIGURE 1:**
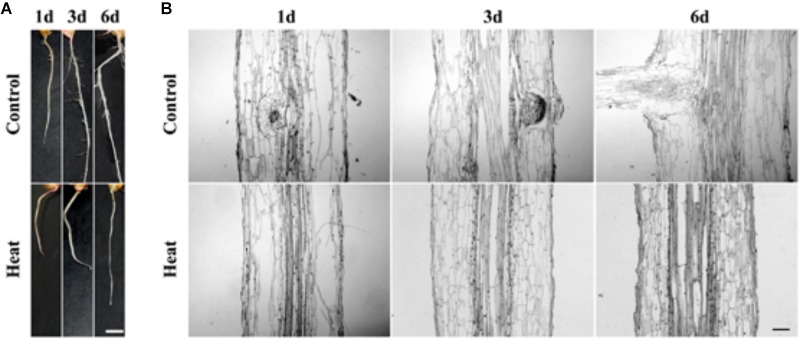
**(A)** Phenotype characteristics of maize plant at 1, 3, and 6 days under heat treatment and control condition. The lateral root primordium (LRP) initiation was inhibited in maize seedling under heat treatment for 1 day hence no lateral root was observed. Bar = 5 mm. **(B)** Paraffin section of the LRP zone. Analysis of paraffin tissue section showed that the LRP initiation was inhibited under heat stress. As compared to the untreated controlled root LRP initiation was not observed under heat treatment incubation at 45°C, suggesting that LRP initiation was inhibited. Bar = 10 μm.

In an attempt to investigate the genetic mechanism of the plant response to heat stress, our results suggested that heat stress led to LRP inhibition in maturation zone of maize seedlings. The seedlings of maize hybrid line *Huayu 5* were treated for 1–6 days at 45°C. No LRP initiation was observed at the individual level in the heat-treated seedlings, whereas several lateral roots appeared in maturation zone of the control roots, showing that heat stress inhibits the LRP formation (**Figure 1A**). To verify the inhibition effects of heat stress on lateral root formation, we performed paraffin section with maturation zones in control and heat treated roots at the individual and tissue level. Our work substantiates that lateral roots were initiated from vascular bundle in control and formation of lateral roots might be hardly observed in heat stressed seedlings (**Figure 1B**). Therefore, our results have shown that heat stress might significantly inhibit the initiation of LRP formation from vascular bundle.

### Genomic Histone Hyperacetylation Accompanied the Inhibition Process

**FIGURE 2:**
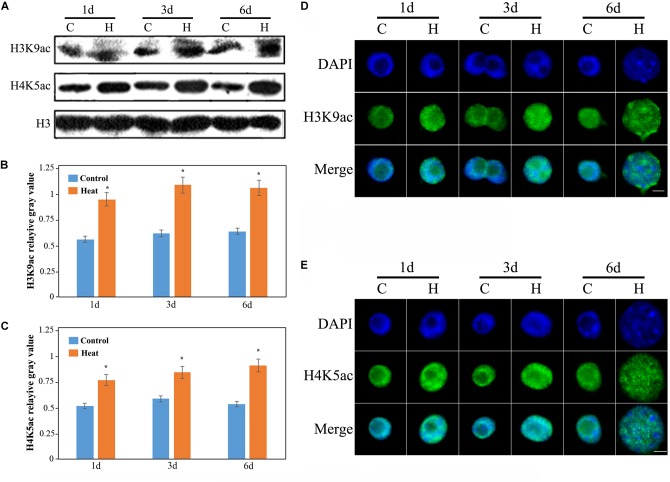
Histone acetylation showed an increasing trend in the heat-induced inhibition of lateral root initiation in maize seedlings. **(A)** The levels of H3K9ac and H4K9ac in the roots of maize seedlings increased significantly under heat treatment. All the western blot assay were repeated three times, and histone H3 was used as the standard internal reference. **(B)** The mean gray value of H3K9ac bands. **(C)** The mean gray value of H4K5ac bands. **(D)** Immunological staining indicated an increase in the acetylation level of H3K9. **(E)** Immunologic staining showed an increase in the acetylation level of H4K5. The level of histone acetylation in the lateral root development of maize seedling was increased, and the nucleus was decondensed. Five hundred nuclei were observed in each sample. The Bar = 10 μm. Asterisk (^∗^) indicated that the gene expression level of the heat treatment group which was found to be significantly different from that of the control group (*t*-test, *p* < 0.01).

To analyze whether heat stress affects histone acetylation in the seedling roots, we have performed western blot analysis with anti-H3K9ac, anti-H4K5ac and anti-H3. H3K9ac and H4K5ac are the two important euchromatic marks positively regulating gene transcription. We have determined that genomic acetylation levels of H3K9 and H4K5 were both significantly increased under heat stress, compared with the untreated control roots (**Figures 2A–C**). Furthermore, we have carried out immunostaining assay to detect histone acetylation at the cellular level. The immunostaining results were consistent with previous results and the distribution of H3K9ac and H4K5ac were found to be unaltered under heat stress as compared with the control (**Figures 2D,E**). Besides, nucleus size was larger under heat stress than that of the control, and possibly linked to the genomic histone hyperacetylation (**Figures 2D,E**).

**FIGURE 3:**
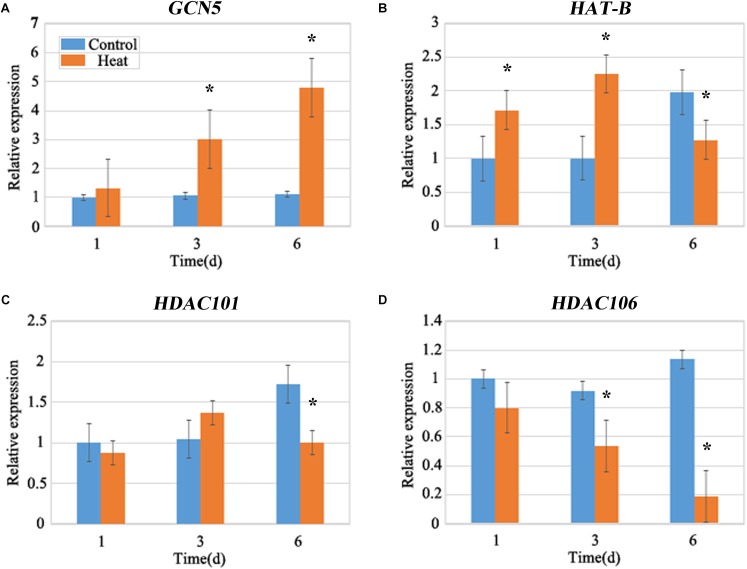
Quantitative Fluorescence PCR analysis. The expression levels of the maize *histone acetyltransferase* (*HATs*), *GCN5* **(A)**, *HAT-B* **(B)**, and *histone deacetylase* (*HDACs*), *HDAC101* **(C)** and *HDAC106* **(D)** under heat stress. The expression of *HATs*, *GCN5* and *HAT-B* were up-regulated, and the expression of *HDACs*, *HDAC101* and *HDAC106* showed a decreasing trend under heat treatment for 1–6 days. The gene expression level of 1 day in the control group was set as 1. Actin is a standardized internal reference y. Each experiment was repeated three times. Asterisk (^∗^) indicated that the gene expression level of the heat treatment group which was found to be significantly different from that of the control group (*t*-test, *p* < 0.01).

Histone acetylation is regulated by *histone acetyltransferases* (*HATs*) and *HDACs* (Kouzarides, 2007). To analyze the expression levels of *HATs* and *HDACs*, two *HAT* genes *GENERAL CONTROL NON-DEREPRESSIBLE 5* (*GCN5*) and *HAT-B* representing two types of *HATs* (*HAT-A* and *HAT-B*), and two HDAC genes (*HDAC101* and *HDAC106*) were investigated. The expression of *GCN5* consistently showed an increased level under heat stress and it was nearly four-fold higher after 6 days of treatment than that in the control group (**Figure 3A**). The expression of *HAT-B* was increased after 1 and 3 days of heat treatments, however it was decreased after 6 days of heat treatment (**Figure 3B**). In brief, the expression of *HATs* were increased under heat treatment. The expression of *HDAC101* exhibited no distinct changes after 1 and 3 days of treatments while it declined after 6 days of treatment (**Figure 3C**). The expression of *HDAC106* revealed a relatively decreased level after heat treatment and it was over 80% lower than that in the control after 6 days of treatment (**Figure 3D**). Overall, the expression of *HDACs* displayed a lower level after heat treatment. The expression changes in *HATs* and *HDACs* genes were consistent with the genomic histone hyperacetylation under heat stress.

### Genomic Histone Hyperacetylation Perhaps Not Be the Key Regulator of LRP Inhibition

**FIGURE 4:**
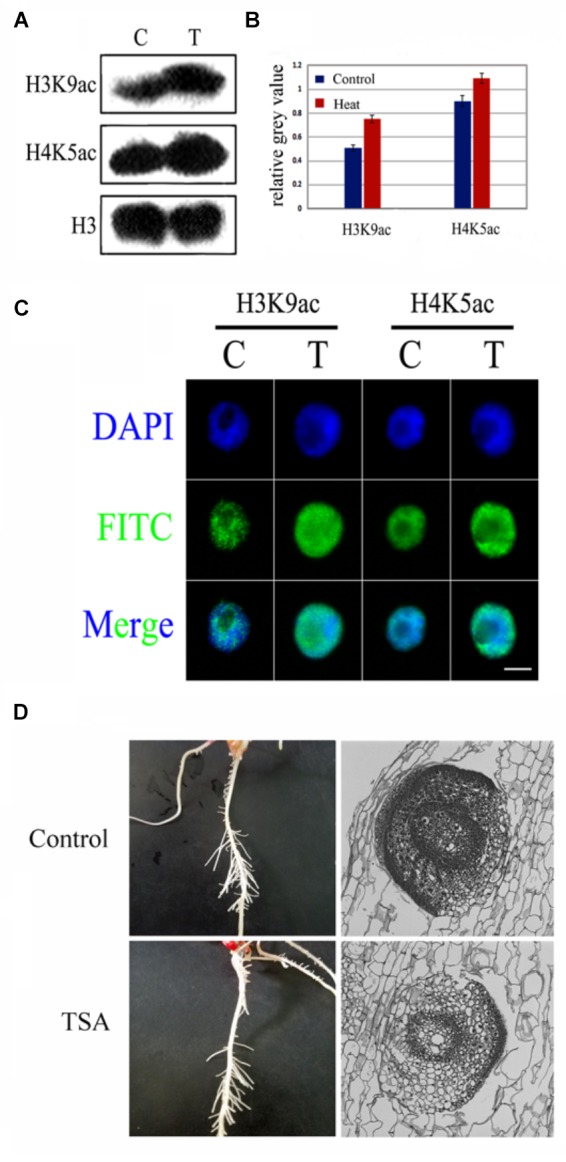
TSA, an epigenetic inhibitor did not inhibit LRP initiation development in maize seedlings. **(A,B)** Western blot analysis showed that H3K9ac and H4K9ac levels in the maize seedlings was significantly increased after TSA treatment. **(C)** The nucleus was decondensed after TSA treatment. **(D)** Phenotype characteristics of maize plants under TSA treatment and control. TSA treatment did not inhibit the LRP initiation in maize, and the paraffin sections of the LRP in roots maize seedling at 3 days under TSA treatment. Western blot experiment is repeated three times. Five hundred nuclei were examined in each sample. Bar = 10 μm.

An increase in histone hyperacetylation under heat treatment led us to investigate the role of histone hyperacetylation in lateral root formation under heat stress. To examine the hyperacetylation level of the genome under heat stress, TSA, an inhibitor of HDAC, was used to treat maize seedlings; both analyses have clearly demonstrated the inhibitory effects. The western blotting results revealed that TSA had clearly enhanced the H3K9ac and H4K5ac levels (**Figure 4A**) and the immunostaining of nuclei further displayed similar results (**Figure 4B**). Notably, TSA might not be involved in the inhibition of LRP formation at both individual and tissue levels (**Figures 4C,D**), suggesting that genomic hyperacetylation perhaps not be the key regulator involved in the inhibition of LRP.

### Heat Stress Inhibits Expression of *HO-1* Gene and *GSL* Gene Family

**FIGURE 5:**
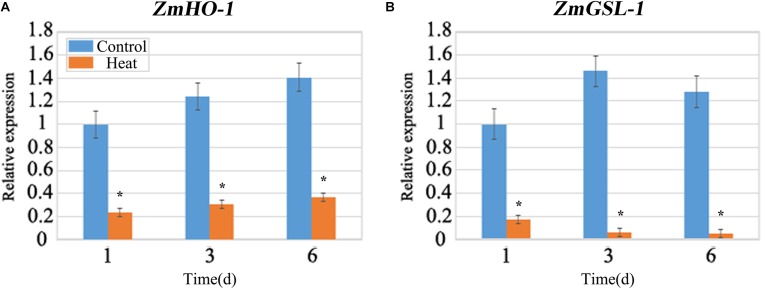
qRT-PCR analysis of the expression levels of *ZmHO-1* and *ZmGSL-1* genes at 1, 3, and 6 days under heat stress. The transcription level of *ZmHO-1* **(A)** and *ZmGSL-1* **(B)** were significantly inhibited under heat stress. The relative expression level was evaluated from three biological replications. Asterisk (^∗^) indicated that the expression level of the heat treatment group was significantly different from that of the control group (*t*-test, *p* < 0.01).

Due to the inhibition of LRP formation under heat stress, so we detected the expression of the related genes involved in lateral root formation. *ZmHO-1* gene was reported to play a role in determining lateral root development and several members of the *GSL* gene family are found to be involved in GA3-regulated lateral root formation. qRT-PCR analysis revealed that the expression of the *HO-1* gene after heat treatment was 70% lower than that under control conditions (**Figure 5A**). Besides, gene expression of *GSL-1* under heat stress was 80% lower than that under control at 1 day and decreasing trend was observed with the increasing days of treatment (**Figure 5B**). We further analyzed the expression patterns of other *GSL* gene family members and our work substantiates that *GSL-4* and *GSL-9* gene expression was similar with *GSL-1* gene expression under heat treatment whereas *GSL-2* and *GSL-6* gene expression was increased after heat treatment (**Supplementary Figure S1**).

### Regulation of *HO-1* and *GSL-1* Gene Expression and Their Association With H3K9ac and H4K5ac on the Promoter Region

**FIGURE 6:**
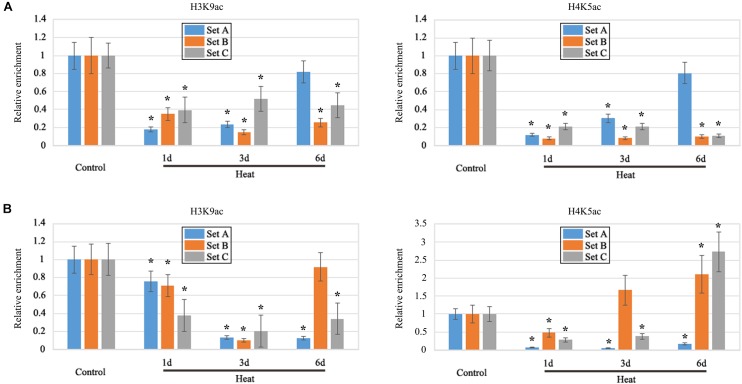
ChIP analysis. It revealed that the modification of H3K9ac and H4K5ac in the promoter region of *ZmHO-1* and *ZmGSL-1* genes was significantly altered under the heat stress. **(A)** The level of H3K9ac and H4K5ac in the upstream region of *ZmHO-1* gene was significantly decreased under heat treatment. **(B)** The level of H3K9ac in the upstream region of *ZmGSL-1* gene was significantly decreased and H4K5ac was found to be slightly present on the set B after 3 days of treatment, whereas it increased significantly on the set C after 6 days of treatment. The acetylation level of the control group was set to 1.0 at each time point. The relative abundance of histone modification was repeated three times. Asterisk (^∗^) indicated that the relative increase of H3K9ac or H4K5ac under heat treatment group is significantly different from that of the control group (*t*-test, *p* < 0.01).

Histone acetylation on the promoter region plays a crucial role in the regulation of gene expression, including H3K9ac and H4K5ac. As, heat stress inhibited the LRP formation therefore, we have focused our study on the specific genes which displayed negative regulation under heat treatment. As a result, we selected two genes, *HO-1* and *GSL-1* to detect histone acetylation levels on the promoter regions; both genes were found to be significantly decreased under heat stress. Furthermore, ChIP assay with antibodies for H3K9ac and H4K5ac were performed (**Supplementary Figure S2**). H3K9ac and H4K5ac were both significantly decreased on three sets of *HO-1* gene under heat treatment as compared to control (**Figure 6A**); most of the differences were more than 50% and the big gap value has been consistent with the previous transcription results as shown by qRT-PCR (**Figure 6A**). H3K9ac was decreased on three sets of *GSL-1* gene under heat treatment, whereas H4K5ac level was fluctuating under heat stress (**Figure 6B**); H4K5ac was found to be slightly present on the set B after 3 days of treatment, whereas it increased significantly on the set C after 6 days of treatment (**Figure 6B**).

### Heat Stress Inhibition of LRP Released in Recovery Group as Accompanied by Release of *HO-1* and *GSL-1* Gene Expression

**FIGURE 7:**
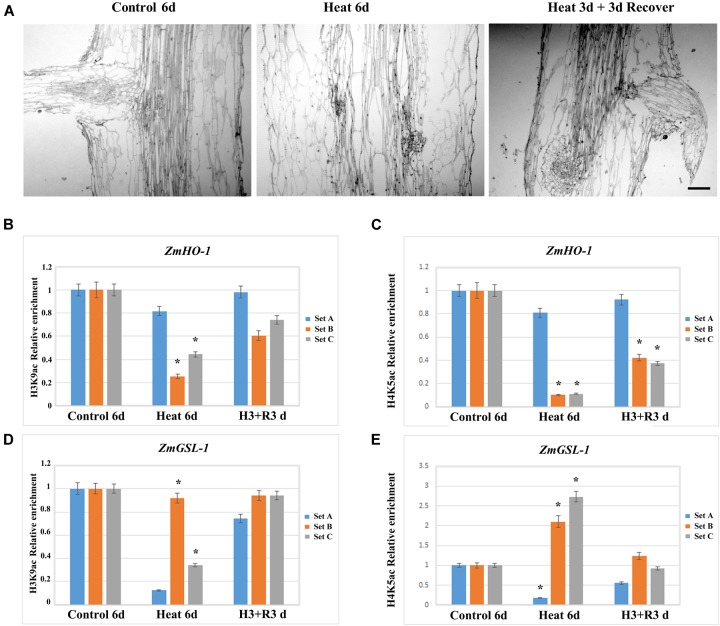
Maize seedlings under heat treatment for 3 days were transferred to the control temperature condition for recovery, and the lateral root growth initiation was restored, while the level of epigenetic modification of *ZmHO-1* and *ZmGSL-1* gene promoter was restored to normal level. **(A)** Root section of maize seedlings at 6 days under control temperature condition and 6 days under heat treatment. **(B)** The alterations in H3K9ac in the upstream region of *ZmHO-1* gene promoter under heat treatment group and restored group. **(C)** The alterations of H4K5a*c* in the upstream region of *ZmHO-1* gene promoter in heat treatment group and restored group. **(D)** Changes in H3K9ac in the upstream region of *ZmGSL-1* gene promoter under heat treatment group and restored group. **(E)** Alterations in H4K5ac upstream of *ZmGSL-1* gene promoter under heat treatment group and restored group. All experiments were repeated three times. Asterisk (^∗^) indicated that the relative increase in H3K9ac or H4K5ac under heat treatment is significantly different from that of the control group (*t*-test, *p* < 0.01).

In order to investigate whether the inhibition of LRP formation occurred under heat stress might be recovered and to elucidate the function of histone acetylation on promoter regions of *HO-1* and *GSL-1* genes, plants after 3 days of treatment were transferred to control conditions for 3 days. The results indicated that lateral roots appeared in the maturation zone of seedling roots after 3 days of recovery (**Figure 7A**). The paraffin section examination at tissue levels further supports the results obtained at the individual level (**Figure 7A**); the expression of *HO-1* and *GSL-1* genes were clearly enhanced as compared with their respective groups under heat stress (**Figures 7B–E**).

### Histone Deacetylation Induces Inaccessibility of the Promoter Region of *ZmHO-1* and *ZmGSL-1* to Micrococcal Nuclease

**FIGURE 8:**
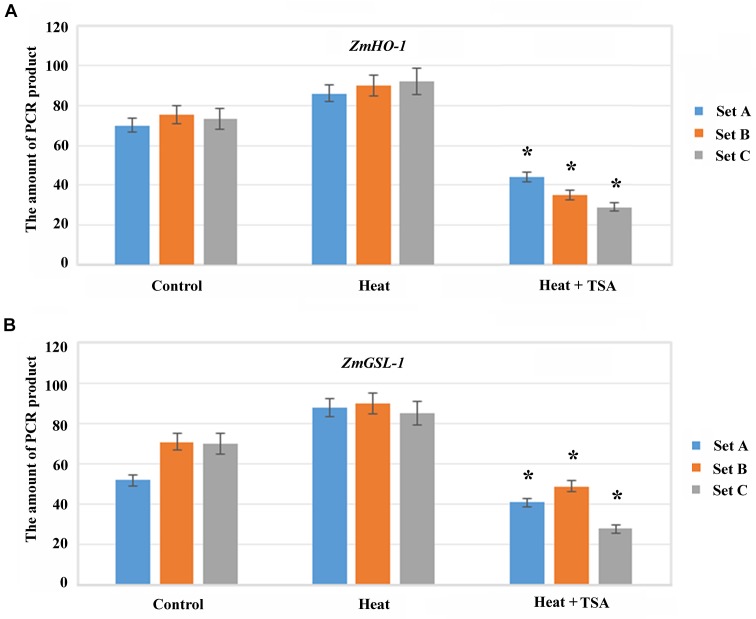
Histone acetylation induced chromatin accessibility of the promoter region in *ZmHO-1* and *ZmGSL-1*. **(A,B)** CHART-PCR assay was performed with nuclei digested by MNase to measure chromatin accessibility of the specific promoter region of *ZmHO-1* and *ZmGSL-1*. The PCR products was converted from the Ct values according to the standard curve. All experiments were repeated three times. Asterisk (^∗^) indicated that the relative PCR product in *ZmHO-1* and *ZmGSL-1* of the TSA treatment group was significantly different from that of the control group (*t*-test, *p* < 0.01).

As, histone acetylation/deacetylation usually alters chromatin conformation to regulate gene expression therefore, we analyzed the effect of TSA on local region chromatin accessibility to micrococcal nuclease (MNase) during heat treatment. Condensed chromatin regions are more inaccessible to MNase digestion as compared to the decondensed chromatin regions, so real-time PCR (CHART-PCR) was used to analyze MNase digestion and chromatin accessibility. By CHART-PCR analysis we investigated the chromatin packaging of the specific region. In heat treated group, set A, B and C were less accessible to MNase (**Figure 8**) as compared to control group. Thus, HDACs might induce the chromatin decondensation across the promoter region during heat treatment, which apparently alter the expression of *ZmHO-1* and *ZmGSL-1*.

## Materials and Methods

### Plant Materials and Heat Stress Treatment

Seeds of maize (*Zea mays*) hybrid line *Huayu 5* were germinated and seedlings were cultured in water for 3 days under untreated controlled environmental conditions (14 h light/120 mmol⋅m^−2^⋅s^−1^/25°C and 8 h dark/20°C, 70% relative humidity) (Yong et al., 2012). Subsequently, plants under heat treatment were incubated at 45°C with the similar photoperiod and humidity. Roots from different treatments were collected and used for different assays. The concentration of TSA used in the present investigation was 10 μM (Wang et al., 2015). In the TSA treated group, the maize seedlings were transferred to the solution of 10 μM TSA and the solution was supplemented with 10 μM TSA per day.

### Fixation and Sectioning

Root samples of the control and under high temperature group for 1, 3, and 6 days were selected and immersed in FAA solution [glacial acetic acid, formalin (37%) and ethanol (70%); 1:2:17 (v/v)] for approximately 24 h at room temperature. Leica TP-1050 tissue processor was used to process fixed root tissues for critical drying and wax infiltration. Rotary microtome was used to cut chilled wax embedded root samples to obtain 8 μm tissue section and then flattened on the surface of a water bath at 42°C. The tissue sections were transferred onto slides by lifting the slides beneath the wax sections. Afterward, the wax sections were melted on a hot plate (∼60°C to be affixed upon the slides). The slides were sequentially processed through de-waxing, staining and mounting after oven-drying at 38°C. The prepared slides were examined using the Olympus BX-60 light microscope (Olympus, Tokyo, Japan) and photographed with the CCD monochrome camera Sensys 1401E.

### Antibodies

The antibodies used in western blotting, immunostaining and ChIP assays were as follows: anti-H3 (ab1791) and anti-H4K5ac (ab1997) were obtained from Abcam; anti-H3K9ac (07-352) and fluorescein-conjugated goat anti-rabbit IgG (16-237) were obtained from Millipore and AP-conjugated goat anti-rabbit IgG (A4187) was obtained from Sigma.

### Western Blot Analysis

Proteins were extracted from the maize seedlings, by grinding the roots in liquid nitrogen and re-suspending the powder in the protein extraction buffer [100 mM Tris-HCl pH 7.4, 5 mM EDTA, 50 mM NaCl and 1 mM PMSF] was performed as previously described (Zhao et al., 2014). Western blot detection was carried out as previously described (Zhang et al., 2011). The mean gray value of the signals of H3K9ac and H4K5ac was measured with ImageJ 1.48 software. Abundance index was calculated as H3K9ac or H4K5Ac band intensity/H3 band intensity. Histone H3 was used as a loading control. All assays were repeated three times.

### Immunostaining Analysis

Nucleus preparation and immunostaining were performed as previously described (Fei et al., 2010). Briefly, isolated nuclei (*n* = 500) were spread on a slide and incubated with the primary antibody at 4°C overnight followed by an incubation at 37°C for 2 h with the secondary antibody. All slides were examined under an Olympus BX60 fluorescence microscope (Olympus, Tokyo, Japan), after counterstained with 0.2 μg/ml DAPI (Sigma), mounted with Vectashield (Vector labs, Burlingame, CA, United States). Images captured with the CCD monochrome camera Sensys 1401E were pseudo-colored using the METAMORPH^®^ 4.6.3 software (Universal Imaging Corp, Downingtown, PA, United States). Microscope settings and camera detector exposure time were kept constant for each respective channel (fluorescein or DAPI) but were optimized for individual experiments. For both control and treatment groups, three independent immunostaining experiments were performed with each antibody.

### Quantitative Real-Time PCR (qRT-PCR) Analysis

Total RNA was isolated from maize roots using the RNAprep pure Plant Kit (Qiagen, Mannheim, Germany) following the supplier’s instructions. To remove residual DNA contamination, 1 mg of total RNA was treated with 50 units of *DNase I* (Fermentas, Burlington, ON, Canada) at 37°C for 30 min. The purified RNA was reverse-transcribed to cDNA using a Revert Aid First Strand cDNA Synthesis Kit (Fermentas, Burlington, ON, Canada).

qRT-PCR was performed using SYBR^®^ Green Real-time PCR Master Mix (Toyobo, Tokyo Japan) in a StepOne Plus real-time PCR system (Applied Biosystems) with the following cycling conditions: 94°C for 2 min, followed by 40 amplification cycles at 94°C for 5 s, 56°C for 15 s and 72°C for 20 s. Fluorescence data were acquired at the 72°C step and during the melting curve program. The *Glyceraldehyde-3-phosphate dehydrogenase* (*GAPDH)* gene (GenBank accession number: X07156.1) was selected as a reference gene in this study. Template-free and SYBR Green mix-free samples were amplified for each gene as negative controls. Triplicate PCR reactions for each of the three independently-purified RNA samples were carried out. Quantitative PCR primers were designed using the Primer Premier 5 software to amplify fragments of approximately 200 bp (**Table 1**).

**Table 1:** Primers used for qRT-PCR.

RT-PCR	Sequence (5′-3′)	Efficiency %
*ZmHO-1*	ACACTGTTGGCTGATCCAGT	96
	AAACGTATCTGGGGGAGGGA	
*ZmGSL-1*	CTAATTTGCTGCGCGGCAATG	98
	CACTTGCGGCAGAAGAAGAG	
*Actin*	GATGATGCGCCAAGAGCTG	102
	CCTCATCACCTACGTAGGCAT	

### Chromatin Immunoprecipitation (ChIP) Assay

ChIP assay was performed with anti-H3K9ac and anti-H4K5ac as previously described (Haring et al., 2007). The immunoprecipitated DNA was subjected to real-time PCR analysis with six primer sets, designated as A–C (**Table 2**) for the *ZmHO-1* and *GSL-1* gene promoter regions following the above-mentioned procedure.

**Table 2:** Primers used for ChIP-PCR and CHART-PCR.

ChIP-PCR	Sequence (5′-3′)	Efficiency %
*ZmHO-1* Set A	CCATACTCGAGCTGCTCA	101
	AGAGGGACATTCAGGGA	
*ZmHO-1* Set B	GATAGTTCCGATGAAGAG	98
	AGTCATCTTCCTCAGACA	
*ZmHO-1* Set C	GGACGGCTGAAGTTTCTCTG	97
	GCTTGCATAAGGGCGATAAG	
*ZmGSL-1* Set A	CAGCTGACCTGATGGAGACT	104
	TTGGCATCTGCAACAGACGC	
*ZmGSL-1* Set B	ACACTGTTGGCTGATCCAGT	96
	AAACGTATCTGGGGGAGGGA	
*ZmGSL-1* Set C	CTAATTTGCTGCGCGGCAATG	98
	CACTTGCGGCAGAAGAAGAG	
*ZmActin*	GATGATGCGCCAAGAGCTG	102
	CCTCATCACCTACGTAGGCAT	

### Chromatin Accessibility Real-Time PCR (CHART-PCR)

CHART-PCR assay was performed to analyze the conformational change of chromatin as previously described (Xu et al., 2016). The seedlings treated with heat or heat-TSA for 1 day. Nuclei were extracted and digested using 5 U MNase for 5 min at 37°C (Hu et al., 2011). Subsequently, DNA was prepared using a Plant genomic DNA kit (Qiagen, Mannheim, Germany) and quantified using the Gene Quant calculator (Amersham Pharmacia Biotec, Piscataway, NJ, United States). 100 mg of genomic DNA from heat or heat-TSA treated samples was used for SYBR Green real-time PCR analysis. The primers used in chromatin accessibility by real-time PCR were similar to those used in the ChIP assays (**Table 1**). MNase accessibility is characterized to be inversely proportional to the amount of amplified PCR product.

## Discussion

In the present study, we have investigated the possible relationship between histone acetylation at the gene promoter regions and LRP inhibition in maize seedling under heat stress. The results provided a new insights into a possible epigenetic regulation of the heat-induced LRP inhibition.

Histone modification plays a vital role in plant response to abiotic stresses to modulate epigenetically the growth and development by remodeling the chromatin structure and activating or repressing gene transcription (Geiman and Robertson, 2002). Maize roots under heat treatment had shown distinct morphological and histological features of inhibited LRP formation. The global acetylation level of histone H3K9 and H4K5 increased significantly under heat treatment, which suggested the potential role of heat stress in histone modification. The increased expression of *GCN5* and *HAT*-*B* and the decreased expression of *HDAC101* and *HDAC106* genes might contribute to the histone acetylation changes in maize seedlings exposed to heat stress. The increased level of histone acetylation consistently leads to an open access of chromatin (Bannister and Kouzarides, 2011). In the present investigation, the increase in global histone acetylation of H3K9ac and H4K5ac accompanied by chromatin decondensation, indicated high accessibility of the whole genome allowing transcription factor recruitment in the process of LRP inhibition after heat treatment and our result were consistent with the histone acetylation and deacetylation study conducted on yeast (Kurdistani and Grunstein, 2003). Furthermore, we investigated the role of histone hyperacetylation in the process of LRP inhibition. TSA, a HDACs inhibitor, was used to treat maize seedlings; our results had shown similar histone hyperacetylation level under heat stress (Wang et al., 2015). The western blotting analysis had indicated that TSA led to enhanced levels of H3K9ac and H4K5ac and the immunostaining of nuclei also suggested similar results in control condition roots as compared to heat stress. As the LRP formation was not inhibited by TSA, further suggesting that global hyperacetylation might not be the determining factor in the inhibition process of lateral root as induced under heat stress.

*ZmHO-1* gene was reported to play a crucial role in lateral root development and several members of the GSL gene family were found to be crucial in GA3-regulated lateral root formation. *HO*-*1* and *GSL*-*1* genes were down-regulated under heat treatment as compared to control condition. After 3 days of recovery period, the expression of *HO*-*1* and *GSL*-*1* genes were increased as compared with the heat stress and LRP initiated in the maturation zone, exhibiting the release of LRP inhibition. Moreover, the expression level of other *GSL* gene family members were also detected using qRT-PCR. *GSL-4* and *GSL-9* gene expression were similar with *GSL-1* under heat stress. However, *GSL-2* and *GSL-6* gene expression levels were increased under heat stress (**Supplementary Figure S1**). Therefore, our result suggested that *ZmHO*-*1* and *ZmGSL*-*1* seems to be involved in the heat induced LRP inhibition in maize.

The GSL gene family *ZmGSL2* is only active in primary roots, while *ZmGSL6* is detected in secondary root. *ZmGSL4* is strongly expressed in wild-type and *lrt1* mutant primary roots but only transcribed at low levels in primary roots of *rum1*. However, *ZmGSL9* is strongly expressed in wild-type primary roots and only weakly transcribed in *lrt1* and *rum1*. In this study, expression level of *GSLs* under heat treatment is different, which might be attributed to their different function in lateral root development.

**FIGURE 9:**
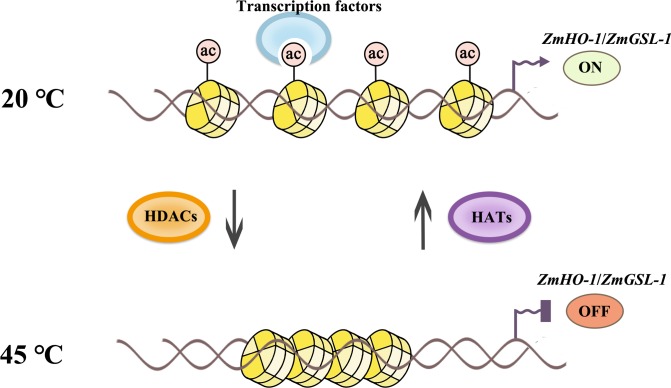
Schematic representation of histone acetylation regulation in lateral root development under heat stress. Histone acetylation level of *ZmHO-1* and *ZmGSL-1* gene promoter region were decreased under heat treatment. The lower acetylated histone apparently highly charged and binds with DNA phosphate backbone closely. The transcription factor and RNA polymerase II might not access DNA, therefore inhibited transcription, subsequently led to the lateral root abnormality in maize.

Histone modifications of chromatin on the promoters can reveal repository information about developmental and environmental cues. Histone acetylation/deacetylation at the promoter regions of some genes is usually involved in the alteration of the local chromatin conformation that regulates gene expression, and our findings were found to be consistent with the previous results. For example, suberoylanilide hydroxamic acid activates the transcription of the *p21WAF1* gene by increasing the levels of histone acetylation in the promoter region (Richon et al., 2000). *Arabidopsis* *HOS15* represses expression of *RD29A* through RD29A promoter-associated histone deacetylation (Zhu et al., 2008). *Arabidopsis* *AtHD2A*, *AtHD2B*, and *AtHD2C* were shown to repress transcription when targeted to the promoter of genes (Wu et al., 2003). In this study, ChIP assay with anti-*H3K9ac* and anti-*H4K5ac* indicated that histone acetylation levels on the promoter regions of *ZmHO-1* and *ZmGSL-1* genes were significantly decreased during heat stress, except for *H4K5ac* level of promoter area of *GSL-1* which was slightly accumulated in the set B after 3 days of heat treatment and certainly increased in the set C after 6 days of treatment. Besides, CHATR-PCR data suggested that the chromatin accessibility of the promoter regions of *ZmHO-1* and *ZmGSL-1* was decreased under heat treatment and increased under heat-TSA treatment, indicating that heat stress inhibits the expression of *ZmHO-1* and *ZmGSL-1* through chromatin alteration in specific sites that regulate expression of these genes (**Figure 9**). Additionally, our results has been consistent with the previously published report on inhibition of *sodCp* genes in response to abscisic acid through deacetylation of histones in the promoter regions in maize (Hou et al., 2015).

In the present investigation, we have suggested the role of promoter associated histone acetylation of *Haem Oxygenase-1* (*HO-1*) and *Giberellic Acid-Stimulated Like-1* (*GSL-1*) genes in heat induced LRP inhibition in maize, further experiments were required to establish the direct role of *ZmHO-1* and *ZmGSL-1* genes expression in the inhibition of LRP formation in maize under high temperature using promoter modified plants. In conclusion, the maize lateral root formation was found to be suppressed under heat treatment and an epigenetic control of expression of the lateral root formation related genes was observed in response to heat stress.

## Author Contributions

LL, SH, and HZ conceived and designed the experiments. HZ, MY, and XZ performed the experiments. LL and HZ analyzed the data. HZ contributed reagents, materials, and analysis tools. LL, HZ, and MG wrote the paper.

## Conflict of Interest Statement

The authors declare that the research was conducted in the absence of any commercial or financial relationships that could be construed as a potential conflict of interest.

## Supplementary Material

FIGURE S1 qRT-PCR detection of *GSL* gene family expression levels under heat stress **(A)** *GSL-2* and **(C)** *GSL-6* gene expression was increased under heat treatment **(B)** *GSL-4* and **(D)** *GSL-9* gene expression was similar with *GSL-1* gene expression in heat stress. The gene expression level in the control group for 1 day was set to 1. Actin is used as a standardized internal reference. Experiments were repeated three times. Asterisk (^∗^) indicated that the gene expression level of the heat treatment group was significantly different from that of the control group (*t*-test, *p* < 0.01).

FIGURE S2 Design of the detected fragment pattern in the promoter region of *ZmHO-1* and *ZmGSL-1* genes (Set A, B, C).
