# Supplementary figures and images for: The Role of Promoter-Associated Histone Acetylation of Haem Oxygenase-1 (HO-1) and Giberellic Acid-Stimulated Like-1 (GSL-1) Genes in Heat-Induced Lateral Root Primordium Inhibition in Maize
Source: Front Plant Sci. 2018 Oct 16;9:1520. doi: 10.3389/fpls.2018.01520 (PMC6232826; doi:10.3389/fpls.2018.01520)

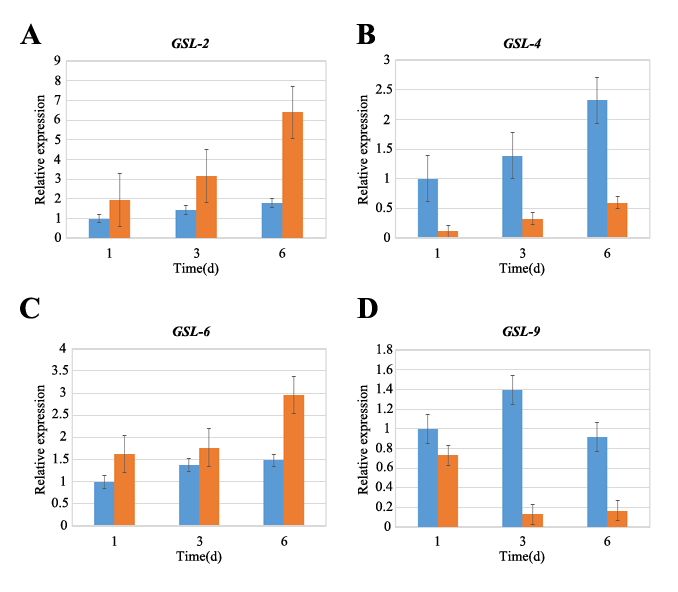

Supplement: FIGURE S1 — qRT-PCR detection of GSL gene family expression levels under heat stress (A) GSL-2 and (C) GSL-6 gene expression was increased under heat treatment (B) GSL-4 and (D) GSL-9 gene expression was similar with GSL-1 gene expression in heat stress. The gene expression level in the control group for 1 day was set to 1. Actin is used as a standardized internal reference. Experiments were repeated three times. Asterisk (∗) indicated that the gene expression level of the heat treatment group was significantly different from that of the control group (t-test, p < 0.01). [file Image_1.TIF]

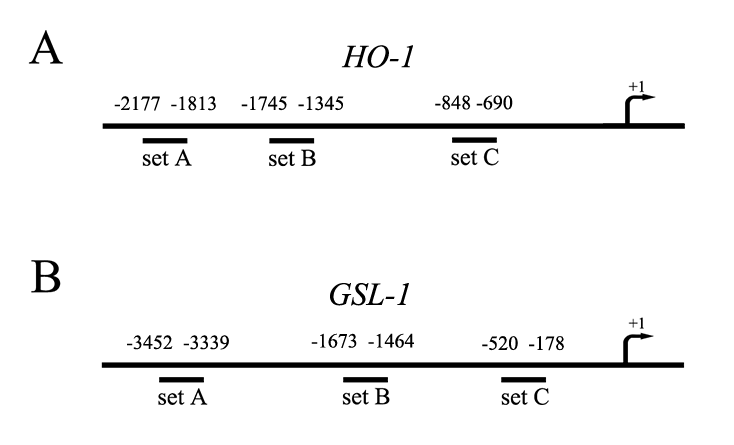

Supplement: FIGURE S2 — Design of the detected fragment pattern in the promoter region of ZmHO-1 and ZmGSL-1 genes (Set A, B, C). [file Image_2.TIF]
